# Development of an objective assessment tool for total laparoscopic hysterectomy: A Delphi method among experts and evaluation on a virtual reality simulator

**DOI:** 10.1371/journal.pone.0190580

**Published:** 2018-01-02

**Authors:** Sophie Knight, Rajesh Aggarwal, Aubert Agostini, Anderson Loundou, Stéphane Berdah, Patrice Crochet

**Affiliations:** 1 Department of Obstetrics and Gynecology, Assistance Publique—Hôpitaux de Marseille, La Conception Hospital, Marseille, Aix Marseille Université, France; 2 Department of Surgery, Thomas Jefferson University,Philadelphia, Pennsylvania, United States of America; 3 Office of Strategic Business Development and Partnerships, Jefferson Health, Philadelphia, Pennsylvania, United States of America; 4 Public Health Laboratory, Aix-Marseille University, Marseille, France; 5 Aix Marseille Université, CERC, IFSTTAR, LBA UMR_T 24, Marseille, France; Indiana University School of Medicine, UNITED STATES

## Abstract

**Introduction:**

Total Laparoscopic hysterectomy (LH) requires an advanced level of operative skills and training. The aim of this study was to develop an objective scale specific for the assessment of technical skills for LH (H-OSATS) and to demonstrate feasibility of use and validity in a virtual reality setting.

**Material and methods:**

The scale was developed using a hierarchical task analysis and a panel of international experts. A Delphi method obtained consensus among experts on relevant steps that should be included into the H-OSATS scale for assessment of operative performances. Feasibility of use and validity of the scale were evaluated by reviewing video recordings of LH performed on a virtual reality laparoscopic simulator. Three groups of operators of different levels of experience were assessed in a Marseille teaching hospital (10 novices, 8 intermediates and 8 experienced surgeons). Correlations with scores obtained using a recognised generic global rating tool (OSATS) were calculated.

**Results:**

A total of 76 discrete steps were identified by the hierarchical task analysis. 14 experts completed the two rounds of the Delphi questionnaire. 64 steps reached consensus and were integrated in the scale. During the validation process, median time to rate each video recording was 25 minutes. There was a significant difference between the novice, intermediate and experienced group for total H-OSATS scores (133, 155.9 and 178.25 respectively; p = 0.002). H-OSATS scale demonstrated high inter-rater reliability (intraclass correlation coefficient [ICC] = 0.930; p<0.001) and test retest reliability (ICC = 0.877; p<0.001). High correlations were found between total H-OSATS scores and OSATS scores (rho = 0.928; p<0.001).

**Conclusion:**

The H-OSATS scale displayed evidence of validity for assessment of technical performances for LH performed on a virtual reality simulator. The implementation of this scale is expected to facilitate deliberate practice. Next steps should focus on evaluating the validity of the scale in the operating room.

## Introduction

Hysterectomy is the second most frequently performed surgical procedure on women, after cesarean section [1]. More than 300 000 inpatient hysterectomies were performed in the US in 2012 [2]. Surgical routes were distributed as follows: abdominal (52.8%), vaginal (14.7%) and laparoscopic (32.4%). Two fifths of laparoscopic hysterectomies performed for benign indications were robotically assisted. The majority of hysterectomies were performed abdominally, though benefits of the vaginal and laparoscopic routes have largely been demonstrated in terms of speedier return to normal activities, lower intraoperative blood loss, reduced wound infections and enhanced cosmetic results [3]. For benign indications, the Cochrane database recommends to favor the vaginal approach over the abdominal approach, and to attempt laparoscopic hysterectomy when vaginal hysterectomy is not possible [3]. Total laparoscopic hysterectomy (LH) has notable advantages over vaginal hysterectomy, such as allowing an optimal exploration of the abdominal cavity. This aspect is particularly interesting in oncological gynecology and for certain benign indications. Furthermore the laparoscopic route offers the possibility to perform additional procedures such as sacrocolpopexy for prolapse treatment or lymphadenectomy in the oncological field. Thus, LH has become the procedure of choice in surgical oncology for treatment of endometrial cancer [4,5].

Although Harry Reich performed the first LH more than 25 years ago [6], this approach is not fully exploited in current gynecologic practice. It has often been suggested that this could be attributed to difficulty to train operators to this procedure [7]. Training remains largely based on companionship in the operating room (OR) [8], although new training frameworks have been developed including a program for LH on a virtual reality (VR) simulator [9]. LH requires an advanced level of surgical skills, with a learning curve estimated between 30 to 75 cases depending on the surgeon’s laparoscopic experience [10,11]. There is no clear definition of proficiency level which is currently based on the number of cases performed as a primary surgeon and on the subjective opinion of a senior preceptor. This method is known to be imprecise and unreliable [12]. Improved training and competence assessment should allow optimal incorporation of this technique into the surgical armamentarium.

Evaluation of technical performance requires objective measurement tools. Generic global rating scales are recognised rating tools but do not provide trainees with information on the specific parts of the procedure that require improvement [13,14]. For this purpose, procedure specific scales have been developed for advanced laparoscopic procedures [15–18]. These procedure specific scales have number of potential applications including assessment of trainee’s operative skills and validation of competences. They can be used to facilitate constructive feedback and deliberate practice.

The objective of the study was to develop an objective scale for assessment of technical skills for LH (H-OSATS) and to demonstrate feasibility and validity in a VR setting.

## Materials and methods

### Hierarchical task analysis (HTA)

A HTA was conducted in order to deconstruct the procedure into its component steps [19]. The purpose was to identify the successive discrete steps that are required to complete a LH. This process can be carried out including the intervention of other members of the team (anaesthetist, scrub nurse). Because the objective of the scale was to focus on surgical technical performances, it was chosen to select steps that referred to the surgeon only.

Literature was searched in order to identify the different operative techniques [20–26]. A panel of video recordings of extrafascial LH performed by expert laparoscopic gynecologists illustrating these techniques were selected from 2 online databases (AAGL, WeBsurg). The aim was to develop a scale that could assess operative performances regardless of the different approaches to the procedure. Two experienced laparoscopic surgeons (AA and PC) reviewed independently the videos. Each reviewer listed all consecutive discrete steps required for completion of each operation. An in-person meeting was organised in order to pool together the results and elaborate a joint list of steps.

### Creation of the H-OSATS scale

The list of consecutive steps generated by the HTA was submitted to a panel of international expert laparoscopic gynecologists using a Delphi method. The Delphi method is a systematic and interactive forecasting method used to obtain consensus among a panel of experts, who are consulted over several rounds. After each round answers are collected, analysed and submitted back in an iterative fashion to the group. Over the successive rounds, group opinion should converge towards consensus [27,28]. Eligible experts were identified based the following criteria: having prior publications on LH; being key opinion leaders in the field of gynecologic laparoscopy; having active involvement among international endoscopic societies. They were invited to participate in the project via email or at the occasion of an international congress. Experts from different geographic zones were recruited (US, Canada, Europe and Australia) in order to develop an internationally relevant scale. Participation to the expert group was voluntary and informed consent was implied if an individual agreed to participate.

Two rounds of the Delphi survey were conducted via an online questionnaire (docs.google.com). Experts were asked to rate, using a Likert scale from 1 “strongly disagree” to 5 “strongly agree”, each discrete step based on its relevance for assessment of operative skills. They were invited to comment their answers in order to modify or add steps during the second round. Mean and standard deviation obtained for each step during the first round were presented to the experts during the second round. A total of three email reminders were sent during both rounds.

A rate of agreement (RoA) was calculated as a measure of consensus among the experts: [(Agreement—Disagreement)/(Agreement + Disagreement + Indifferent)] x 100

A RoA greater than 70% was chosen as a measure of consensus [29,30]. In case of missing data, RoA was calculated by replacing the missing answer by 3, mean and mode. Steps that reached consensus during the second round were included into the final scale. Finally, a numerical scoring scale ranging from 1 to 5 was assigned to each selected step and an arbitrary description of the attribution of points was established [18]. Steps that are performed bilateraly were to be rated for each side.

### Determination of feasibility of use and validity evidence of the H-OSATS scale

This part of the study used a single blinded observational study design. Video recordings of LH performed on a VR simulator (LAP Mentor^TM^ VR; Simbionix-3D Systems, Cleveland, Ohio, USA) were collected. The simulator provides with a LH program that includes bilateral salpingooophorectomy. This program begins after trocar insertion and once the uterine manipulator is in place, and ends after circumferential colpotomy. Patient positioning, trocar insertion, vaginal vault and skin closure could therefore not be assessed. This program displayed good validity evidence during a previous study according to quantitative and qualitative parameters [9].

LH performed by three groups of operators of different levels of experience were evaluated: novices, intermediates (had performed 2–10 LH) and experienced (had performed over 100 LH). A written informed consent was be obtained from each operator. The group of novices comprised 5th year medical students who had spent 3 months rotation in the gynecology OR and assisted at least one LH. Two LH were performed in each group: it was chosen to assess the second LH. Each video was recorded using FRAPS real time video capture software (Beepa Pty Ltd, Melbourne, Victoria, Australia). Videos were anonymized and assessed independently by two trained raters (PC and SK) using the H-OSATS scale and the Objective Structured Assessment of Technical Skills generic global rating scale (OSATS). Due to difficulty in differentiating the 2 last components of the OSATS scale on videos (‘‘Flow of operation and forward planning” and ‘‘Knowledge of specific procedure”), they were evaluated as a single component giving a score out of 30 instead of 35 [13,31].

#### ➢ Feasibility

Raters were asked to record the time required to score LH performed on the simulator, using the H-OSATS scale.

#### ➢ Validity evidence

The contemporary meaning of validity is a unitary concept with multiple aspects that considers construct validity as the whole of validity [32–34]. The following aspects of construct validity were evaluated: evidence of content was ensured by agreement among the Delphi panel. Evidence to support relationship to other variables was provided by comparing scores between 3 groups of different levels of experience. Another aspect of relationship to other variables was evaluated by correlating total scores obtained with the H-OSATS scale with those obtained with the OSATS scale. Evidence of internal structure was provided by evaluating reliability of the H-OSATS: Inter-rater reliability was assessed by correlating total and component scores between 2 independent raters using the intraclass correlation coefficient (ICC: 2-way mixed-effects model, absolute agreement). Test-retest reliability was assessed by correlating scores for the 10 first videos assessed at two different time points by the same rater, using the ICC. Two aspects of validity of Messick’s framework could not be evaluated in this study: response process and consequences.

### Statistics

Data was analysed using SPSS version 20.0 (SPSS, Chicago, Illinois, USA). Results were reported as median values and a level of p < 0.05 was considered statistically significant. Comparison of scores was undertaken using non-parametric tests, Kruskal-Wallis and Mann-Whitney U tests. Correlation between both scales were analysed using Spearman’s coefficient.

Ethical approval was obtained from the Institutional Review Board of the French College of Obstetricians and Gynecologists (CEROG 2015-GYN-0801).

## Results

### Hierarchical task analysis

Eight video recordings of LH performed by expert laparoscopic gynecologists were reviewed and a total of 69 consecutive steps were identified. As the videos collected recorded the intra-abdominal camera view, certain aspects of the full procedure were not visualized. Therefore the reviewers listed steps evaluating patient positioning, abdominal access and skin closure without video support.

This list of steps was distributed into 14 tasks: from “patient positioning” (task 1) to “port removal and skin closure” (task 14). Seven additional points on the order in which tasks should be performed were addressed and were added to the list of steps.

### Delphi process

The 76 steps were submitted to a panel of experts. 14 of the 17 experts who accepted to participate in the project completed the first round of the Delphi process. The composition of the panel of experts is detailed in Table 1. A total of 20/76 steps did not reach the predefined level of consensus. 22 discrete steps were reformulated based on comments made by the experts, 2 steps were added and 2 were deleted and a second questionnaire was developed. This questionnaire was submitted to the 14 experts who had completed the first round and all of them completed the second round. The Delphi survey was conducted between April and November 2015. 12/76 steps did not reach consensus level during the second round. Results are detailed in Table 2.

**Table 1:** Composition of the international expert panel for the online Delphi questionnaire.

	Participants in round 1		Participants in round 2	
Location	Contacted	Responded	Contacted	Responded
Australia	1	1	1	1
Belgium	3	3	3	3
Canada	1	1	1	1
France	2	2	2	2
Italy	2	2	2	2
The Netherlands	1	1	1	1
Turkey	1	0	0	0
UK	1	1	1	1
USA	5	3	3	3
Total	17	14	14	14

**Table 2:** Agreement among panel members for 1st and 2nd round of the online Delphi questionnaire on the list of surgical steps generated by the hierarchical task analysis. Reformulations between round 1 and 2 are underlined, and deletions are in italic.

Tasks	Steps		RoA* 1rst round	RoA 2^nd^round
**1. Patient positioning**	Legs spread apart (with very little flexion from the abdomen)		93%	100%
	Both arms tucked along side		86%	79%
	Buttocks close (slightly over the edge of the operating table) *to the edge of the operating table*		93%	100%
**2. Abdominal access**	Achieve intraperitoneal access using a recognized method (Veress needle, open technique, etc.)		100%	100%
	If open technique: Check the optical viewing trocar is placed in the peritoneal cavity before insufflation		21%	57%
	Create appropriate pneumoperitoneum		100%	100%
**3. Inspection of the peritoneal cavity**	Perform diagnostic laparoscopy (including liver and diaphragm)		93%	93%
	Patient put in *steep* Trendelenburg position allowing appropriate exposure		71%	100%
**4. Trocar insertion**	Avoid epigastric vessels		100%	100%
	Insertion of three operating trocars		71%	79%
	Ergonomic trocar placement		100%	100%
	Look for injuries from port placement		100%	93%
**5. Inspection of the pelvis**	Expose pelvis: retract small bowel and sigmoid colon, perform adhesiolysis if necessary		93%	100%
	Inspection of uterus and adnexas		93%	92%
	Insert the uterine manipulator		93%	79%
	Check that uterine manipulator allows appropriate exposure (i.e: it has its 6 degrees of freedom)		64%** 71% 71%	79%
	Check access to pouch of Douglas and sub-ovarian fossas		86%	92%
	*Visualise ureters as they cross over the iliac vessels and travel downwards to the lateral pelvic walls* Check ureter’s path in the pelvis		92%	100%
**6. Division of the round ligaments**	Manipulator: push uterus cranially and laterally towards the opposite side, *and maintain in a medial position*		78%	93%
	Put round ligament into moderate tension		71%	57%
	Coagulation and transection of the round ligament		78%	86%
	Individualize the front and back fold of the anterior leaf of the broad ligament		78%	71%
**7. Division of the infundibulo-pelvic ligament or the utero-ovarian ligament**	Manipulator: push uterus cranially, laterally towards the opposite side *while being maintained in a medial position*		78%	100%
	Expose IP ligament or utero-ovarian ligament		93%	100%
	If fenestration of the broad ligament is performed	Open the anterior leaf of the broad ligament backwards (parallel with the infundibulo-pelvic ligament)	50%	50%
		Expose the posterior leaf of the broad ligament in its grey area	57%** 64% 64%	86%
		Open a peritoneal window in the broad ligament and enlarge	71%	71%
		Check the ureter has been put at a distance	78%	78%
	If fenestration of the broad ligament is not performed	Identify the ureter by transperitoneal visualisation	64%** 71% 71%	78%
	Coagulate the IP ligament (if adnexectomy) or the utero-ovarian ligament (if interadnexal hysterectomy) using an appropriate energy source or suture		93%	86%
	Section the IP ligament (if adnexectomy) or the utero-ovarian ligament (if interadnexal hysterectomy)		86%	86%
**8. Creation of the bladder flap**	Manipulator: push uterus cranially, towards the opposite side *while being maintained in a medial position or pushed posteriorly*		93%	93%
	*Apply tension to the external fold of the prevesical peritoneum in order to open the plan*		50%	-
	*Open of the vesicouterine plan on the lateral side*		50%	-
	Open the anterior fold of the broad ligament on both sides down to the level of the vesico-uterine reflexion		-	79%
	Manipulator: push uterus cranially and posteriorly *while being maintained in a median position in the transverse plan*		85%	86%
	Section of the anterior peritoneum down to the lower uterine segment		85%	86%
	The bladder is grasped at the midline, applying an anterior-superior traction		50%** 64% 64%	71%
	Opening of the vesicouterine space at the midline *to expose the cervicovaginal margin* until the cervico-vaginal margin is exposed		57%** 64% 64%	79%
**9. Opening of the posterior peritoneum**	Manipulator: push uterus anteriorly and cranially, towards the opposite side, *while being kept in a medial position or pushed anteriorly*		71%	93%
	Dissection and section of the posterior leaf of the broad ligament downwards and towards the insertion of the utero-sacral ligaments on each side		77%	93%
**10. Division of the uterine vessels**	Manipulator: push uterus towards the opposite side, cranially *while being kept in a medial position*		92%	100%
	Optimize exposure of the uterine vessels		100%	100%
	Skeletonize uterine vessels at the ascending portion of the uterine artery		64%** 71% 71%	100%
	If anatomy is not distorted: Identify the ureter prior to division of the uterine vessels		42%	50%
	If anatomy is distorted: Identifying the ureter prior to division of the uterine vessels		100%	93%
	Coagulate the uterine vessels using an appropriate energy source or suture		93%	100%
	Section uterine vessels *at the appropriate level* in the ascending portion, at the level of the colpotomizer		78%	71%
	Divide *the distal* cervical attachment of the cardinal ligament		64%	85%
	Divide the uterosacral ligament		21%	36%
**11. Colpotomy:**	Manipulator: push uterus cranially		92%	100%
	Identify the cervico-vaginal delineation from the colpotomizer		93%	100%
	Check that there are no interposed elements around the vaginal fornices and complete dissection if necessary		78%	100%
	Identify the ureter prior to proceeding to the colpotomy		-14%	36%
	Proceed to circumferential colpotomy using an appropriate energy source		93%	100%
**12. Uterus retrieval and vault closure**	Specimen retrieval vaginally *or laparoscopically by morcellation*		71%	100%
	If the specimen is not retrieved in once piece through vaginal route, this step will not be assessed.			
	Occlude vagina to restore pneumoperitoneum		78%	86%
	Identify the ureter prior to proceeding to the suture		14%	43%
	Suture the vaginal vault angles separately		0%	43%
	Suture the *remaining* vaginal vault with interrupted or continuous sutures		93%	93%
	Vaginal suture including sufficient width of vaginal mucosa and fascia		93%	100%
	Suture includes the US ligaments *to restore pericervical ring* for pelvic support		50%	71%
**13. Haemostasis and inspection**	Irrigation and aspiration of the pelvis		78%	93%
	Check vascular pedicles, bladder reflection and vaginal cuff *if needed* under low abdominal pressure: secure hemostasis is needed		100%	86%
	Check there is no damage to surrounding structures		84%	93%
	Perform cystoscopy or Indigo Carmin test if ureteral integrity is of concern.		-	85%
	Perform cystoscopy or bleu test if bladder integrity is of concern		71%	86%
**14. Port removal**	Remove trocars under direct visualisation and inspect port sites for haemostasis		78%	79%
	Evacuate pneumoperitoneum		84%	100%
	Suture fascia for trocars ≥ 10 mm		93%	100%
	Close skin incisions with any acceptable method		93%	85%
**Order in which tasks should be performed**	Perform tasks 1 (patient positioning) to 5 (inspection of the pelvis) in a chronological order		78%	100%
	Perform task 6 (division of the round ligament) and 7 (division of the IP ligament/utero-ovarian) for each side in any order		21%	57%
	Perform task 8 (creation of the bladder flap) and 9 (opening of the posterior peritoneum) for each side in any order		7%	57%
	Perform tasks 10 (division of the uterine vessels) to 14 (port removal) in a chronological order		64%	86%
**Penalty if tasks performed in an incorrect order**	Errors: task 9 (opening posterior peritoneum) before task 7 (IP ligament or utero-ovarian section)		36%	21%
	Errors: task 10 (division of the uterine vessels) before task 8 (creation of the bladder flap) AND 9 (opening posterior peritoneum) are performed		21%	57%
	Errors: task 11 (colpotomy) before task 10 (division of the uterine vessels)		57%	86%

* RoA: Rate of agreement

** Missing data replaced by 3, mean and mode, respectively

### H-OSATS scale

A total of 64/76 steps were selected for inclusion into the final H-OSATS scale. Thus H-OSATS maximum score for the full scale was 370 points. Steps that are not applicable in most cases, i.e. “If anatomy is distorted: Identify the ureter prior to division of the uterine vessels” were assigned a negative score. The full scale is presented in supporting information (S1 Table).

### Determination of feasibility of use and validity evidence of the H-OSATS scale

A total of 26 video recordings were reviewed and scored: 10 LH performed by novices, 8 by intermediates and 8 by experienced operators. Steps evaluated on the VR program ranged from “round ligament division” to “colpotomy”, except for division of cardinal ligaments which cannot be distinguished from the vaginal cul-de-sac on the VR program, giving a total score out of 210 points.

#### ➢ Feasibility

Median operative time for the novice, intermediate and experienced group were 34, 27 and 16 minutes respectively (p<0.001) Median time to rate each video recording was 25 minutes (37, 32 and 20 minutes for the novice, intermediate and experienced group respectively).

#### ➢ Validity evidence

There was a significant difference between the novice, intermediate and experienced group for total H-OSATS scores (133, 155.9 and 178.25 respectively; p = 0.002). Significant differences in scores were observed for the following components of the scale: task 7 (division of the infundibulo-pelvic ligaments), task 8 (creation of the bladder flap), task 9 (opening the posterior peritoneum), task 10 (division of uterine vessels) and task 11 (colpotomy) (Table 3). Regarding task 8 (creation of the bladder flap), scores for the intermediate group were, in absolute value, higher than those of the experienced group. A post hoc analysis found a significant difference between novices and intermediates and between novices and experts (p = 0.026 and p = 0.006 respectively). There was a significant difference between the novice, intermediate and experienced group for total OSATS scores (15, 21.75 and 26 respectively; p<0.001).

**Table 3:** Comparison of scores for different components of total laparoscopic hysterectomy performed on a virtual reality simulator, as assessed by Hysterectomy Objective Structured Assessment of technical Skill scale, using case-volume criteria for definition of novice intermediate and experienced operators.

Tasks		Novices n = 10	Intermediates n = 8	Experts n = 8	p	Maximum possible score
6	Division of the round ligaments	22.75	24.25	25.75	0.091	30
7	Division of the IP ligament or the utero-ovarian ligament	28.75	36.60	42.5	0.001	50
8	Creation of the bladder flap	26.75	34.75	32.25	0.035	40
9	Opening of the posterior peritoneum	10.75	13.25	17	0.049	20
10	Division of the uterine vessels	34	36.75	46	0.004	50
11	Colpotomy	11.5	16.50	18	<0.001	20
	Total	133	155.90	178.25	0.002	210

Inter-rater reliability for total H-OSATS score was ICC = 0.930 (p<0.001). Inter-rater reliability coefficients for individual tasks ranged from 0.717 (p<0.001) for task 9 (opening of the posterior peritoneum) to 0.940 (p<0.001) for task 7 (division of the infundibulo-pelvic ligaments). Test retest reliability for total H-OSATS scores was ICC = 0.877 (p<0.001).

**Fig 1:**
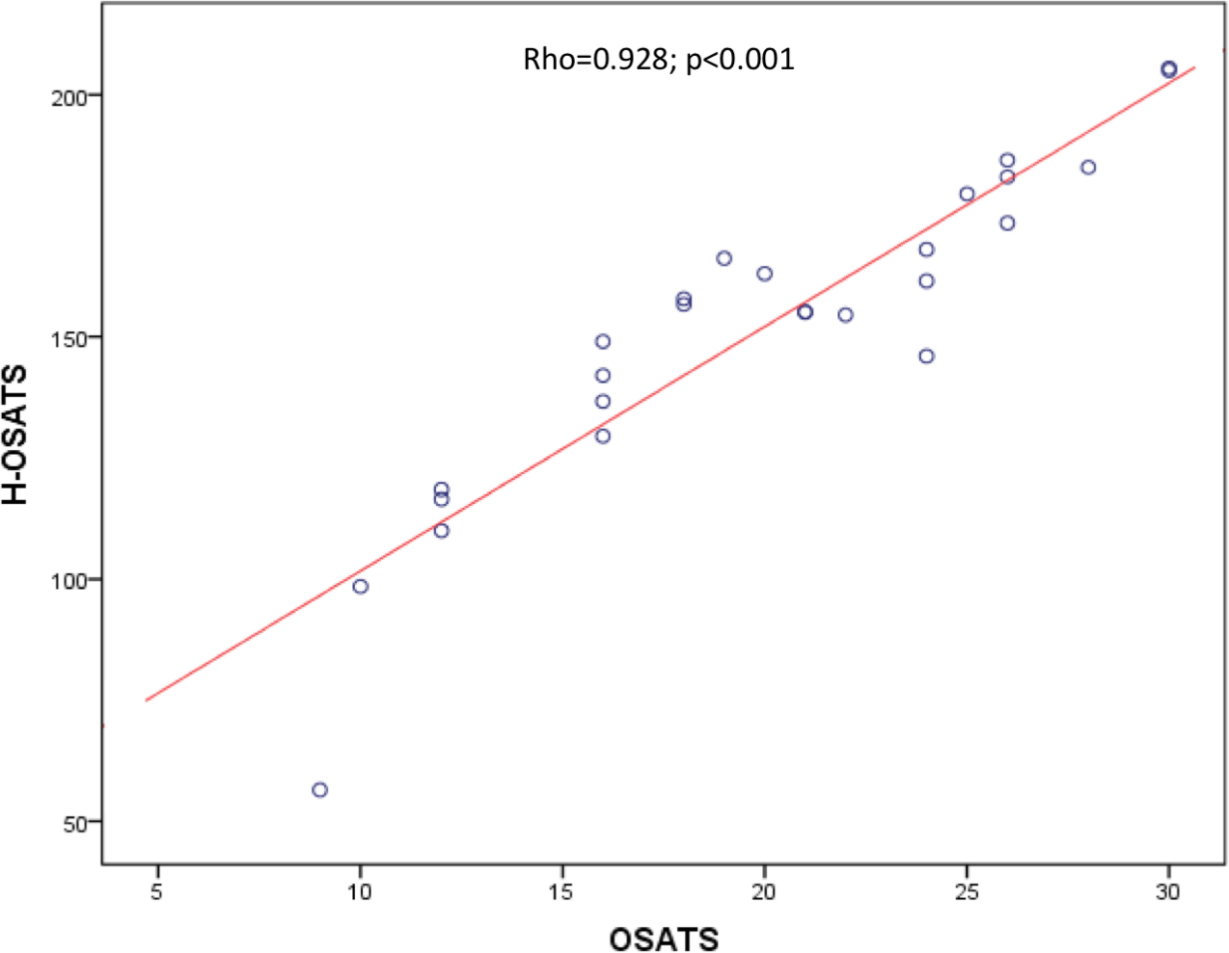
Spearman’s rank correlation between H-OSATS scores and OSATS scores for virtual reality laparoscopic hysterectomy. H-OSATS, Hysterectomy Objective Structured Assessment of Technical Skill; OSATS, Objective Structured Assessment of Technical Skills Global Rating Scale.

A correlation was found between H-OSATS and OSATS total scores (rho = 0.923; p<0.001) (Fig 1).

## Discussion

This study enabled the development of a scale for objective assessment of operative performances for LH, using a systematic approach. The H-OSATS scale is composed of three main parts: patient positioning and exposure (task 1 to 5), the core part of the procedure (tasks 6 to 12) and closure (task 13 and 14). Feasibility of use and sources of evidence to support validity of the H-OSATS scale were demonstrated for the core part of the procedure using a high-fidelity VR laparoscopic simulator.

The advantages of the use of a Delphi method in order to obtain consensus among experts are well described: the anonymous nature of the process prevents a dominant member of the group from influencing the group’s opinion. Furthermore the questionnaire is completed by email and does not require for the experts to physically meet, allowing members from different geographic zones to participate. There is no clear recommendation on the most suitable method to measure a Delphi consensus [35]. Von der Gracht et al. suggested that a RoA is an appropriate measure of consensus particularly when Likert scales are used [36]. The notable limitations of the Delphi method include the fact that the selection of questions submitted to the experts are in part controlled by the Delphi facilitators and that interest of experts can diminish with consecutive rounds. A two round Delphi was conducted in order to limit the number of non-responders and to avoid forced consensus [35].

There are two types of procedure specific assessment tools available: scales and checklists; both these tools have the advantages of identifying specific areas of the procedure that require improvement. Rating scales tend to be less rigid than checklists that oblige operators to perform a series of steps in a predefined order [37]. H-OSATS belongs to the rating scale category. Although these scales are intented to be relatively flexible regarding the chronological sequence of steps, the notion of order in which the main tasks are to be performed was addressed during the Delphi survey. Regarding the core part the procedure, the only point for which a consensus was obtained was that “colpotomy” should not be started before the completion of “uterine vessels division”.

The ability to optimize the role of assistants during laparoscopic procedures is an important component of surgical competency [24]. All steps relating to management of the uterine manipulator were included into the scale, confirming that it is a key element for optimal exposure and closely linked to operative safety [38]. Four other steps regarding exposure were selected. Thus, the use of assistant is considered an operative skill all along the successive parts of the procedure. Some steps that did not reach consensus illustrate the difficulty to standardize some parts of the procedure whose approach can differ depending on surgeons surgical habits but also on the anatomical presentation. For example two steps evaluating fenestration of the broad ligament did not reach consensus during the first round and it appeared from the experts comments that those who usually do not fenestrate the broad ligament rated these steps poorly. Experts were reminded during the second round that the objective was to select steps that best assess operative performances and that the scale should allow assessment of operators regardless of the chosen approach. Therefore, an optional assessment of this part of the procedure was included in the scale. Another issue concerned the uterine vessels division. The level at which the uterine vessels should be coagulated was left with a vague description, as this step often varies depending on the anatomy and operative findings, and reached a high consensus from the first round. However the formulation regarding the level at which the uterine artery should be sectioned was modified based on repeated expert’ comments that encouraged a precise description. The formulation “in the ascending portion, at the level of the colpotomizer” reached consensus at the second round. “Division of the utero-sacral (US) ligaments” did not reach consensus among the experts. There was however a consensus regarding the need to include US ligaments into the vaginal cuff suture. This can be explained by the fact the insertion of these ligaments on the posterior vaginal cul-de-sac remains in most cases. However, the panel of experts considered important that when US ligaments are divided they should be sutured to the vaginal cuff to strengthen pelvic support.

Validity evidence refers to data collected in order to assign a meaningful interpretation of assessment scores [39]. Messick identifies five sources of evidence to support validity: content, response process, internal structure, relationship to other variables and consequences [40]. The H-OSATS displayed good validity evidence with regards to the three tested sources of evidence. The VR setting allowed evaluation of novices in safe conditions, with no intervention from a senior surgeon that could influence results; thus avoiding the inherent bias of operating room (OR) assessment of supervised inexperienced operators. The H-OSATS scale was construct valid for each individual task of the scale assessed by the VR simulator except for task 6 i.e. “division of the round ligaments”. This is probably due to the fact that this task does not require a high degree of technical skills and is relatively easy to perform on the simulator.

Regarding feasibility, average time to assess each video was 25 minutes. This relatively short time can be explained by the fact that the program reproduces a relatively simple case and does not include port insertion and vaginal cuff suture. As operative time for LH reported in clinical studies appears to be longer than operative time performed on the simulator [7], a close look at the time necessary to assess LH using this detailed H-OSATS scale in the OR will be crucial in terms of feasibility. Furthermore, feasability will be evaluated for the full procedure and for each individual task, as in the OR trainees often perform only part of the procedure.

Two other procedure specific scales have been developed for LH. Tremblay et al. published a scale developed from a list of steps arbitrarily chosen with no preliminary HTA and included items that are usually used in generic global rating scales such as « use of assistant to facilitate exposure » [41]. There was no assessment of the validity of this scale, neither in a simulated setting nor in the OR. Frederick et al. recently published an assessment scale for robotically-assisted laparoscopic hysterectomy [42]. Consensus was obtained between 5 experts, based on a list of arbitrarily chosen steps. This study included a validation process in the OR that found significant difference between experts, advanced beginners, and supervised novices. Median scores were surprisingly close (75.6 vs 71.3 vs 69, out of 80), due to the interference of OR supervision according to the authors. Finally, Weizman et al. developed a checklist for laparoscopic suturing of the vaginal cuff using a boxtrainer model [43].

The very detailed nature of the H-OSATS scale makes it an interesting tool for training purposes. H-OSATS can be used in direct observation or video evaluation depending on the chosen training strategy. By identifying specific areas of the procedure that require improvement, H-OSATS should facilitate constructive feedback and thus deliberate practice. It may also be used for research in surgical education. Additional studies are required to explore its potential for use in summative assessment, and to generate specific proficiency cut-off values.

This study has limitations. The H-OSATS scale was constructed in the prospect of a use in the OR. Validity of the entire scale needs to be confirmed in real operative conditions. The VR simulator did not enable the evaluation of tasks 1 to 5 and tasks 12 to 14. However, tasks that are the most specific of this procedure were evaluated, with the exception of vault closure. Operators were allocated to a group based on case volume criteria, although experience does not necessarily correlate with expertise. Comparison of OSATS scores obtained suggested the correct attribution to each group.

This study demonstrated that the H-OSATS scale is a valid instrument for assessment of technical performances for LH performed on a VR simulator. It is a step towards a more objective assessment of technical performances for this advanced laparoscopic procedure. Future research will focus on evaluating the H-OSATS scale in the OR.

## Supporting information

S1 Table H-OSATS scale. (DOCX)
