# Supplementary material for: Development of an objective assessment tool for total laparoscopic hysterectomy: A Delphi method among experts and evaluation on a virtual reality simulator
Source: PLoS One. 2018 Jan 2;13(1):e0190580. doi: 10.1371/journal.pone.0190580 (PMC5749811; doi:10.1371/journal.pone.0190580)
Supplement: S1 Table — (DOCX) [file pone.0190580.s001.docx]

| **Tasks** | | **Steps** | | **1** | **2** | **3** | **4** | **5** | **Score** | |
| --- | --- | --- | --- | --- | --- | --- | --- | --- | --- | --- |
|  |  |  |  |  |  |  |  |  |  |  |
| **1** | **Patient positioning (once patient fully equipped with Foley catheter inserted)** | 1.1 | Legs spread apart (with very little flexion from the abdomen) | Inadequately performed |  | Performed adequately (access to the perineum is not optimal) |  | Performed adequately (optimal access to the perineum) | /5 | /15 |
|  |  |  |  |  |  |  |  |  |  |  |
|  |  | 1.2 | Both arms tucked along side | Not performed |  |  |  | Performed adequately, allowing an easy access to the patient | /5 |  |
|  |  | 1.3 | Buttocks slightly over the edge of the operating table | Not performed adequately |  | Performed adequately, moderate sliding up while in tredelenburg position |  | Performed adequately | /5 |  |
| **2** | **Abdominal access** | 2.1 | Achieve intraperitoneal access using a recognized method (Veress needle, open technique, etc) | Performed with difficulty or inadequately |  | Performed adequately with hesitation but in a safe fashion |  | Performed efficiently and skillfully | /5 | /10 |
|  |  | 2.2 | Create appropriate pneumoperitoneum | Failed to perform |  | Performed with an incorrect pressure and slow to correct |  | Performed efficiently and skillfully | /5 |  |
| **3** | **Inspection of the peritoneal cavity** | 3.1 | Perform diagnostic laparoscopy (including liver and diaphragm) | Not performed |  | Moderately detailed inspection, not all 4 quadrants visualized |  | Careful and thorough inspection (including liver and diaphragm) | /5 | /10 |
|  |  | 3.2 | Patient put in Trendelenburg position allowing appropriate exposure | Not performed |  | Performed after delay. Suboptimal exposure |  | Patient sufficiently tilted allowing optimal exposure | /5 |  |
|  |  |  |  |  |  |  |  |  |  |  |
| **4** | **Trocar insertion** | 4.1 | Avoid epigastric vessels | Location of the epigastric vessels not checked |  |  |  | Anatomical landmarks identified + transillumination performed | /5 | /20 |
|  |  | 4.2 | Insertion of 3 operating trocars | Performed inadequately; without intraperitoneal visualization |  | Performed adequately in a safe fashion |  | Performed smoothly and skillfully | /5 |  |
|  |  | 4.3 | Ergonomic trocar placement | Port placement does not allow appropriate triangulation |  | Port placement allows triangulation, but awkard posture of the surgeon |  | Port placement allowing appropriate triangulation and ergonomic operator posture | /5 |  |
|  |  | 4.4 | Look for injuries from port placement | Not performed |  | Injury cannot be definitively excluded |  | Injury properly excluded | /5 |  |
| **5** | **Inspection of the pelvis** | 5.1 | Expose pelvis: retract small bowel and sigmoid colon, perform adhesiolysis if necessary | Failed to expose pelvis adequately; use of traumatic grasper; adhesiolysis causing damage to surrounding structures |  | Exposed pelvis clumsily. Adhesiolysis performed adequately with some hesitation |  | Pelvis exposed adequately and smoothly using an atraumatic grasper | /5 | /30 |
|  |  | 5.2 | Inspection of uterus and adnexas | Not performed |  | Inspection done but some areas not adequately visualized |  | Careful and thorough inspection | /5 |  |
|  |  | 5.3 | Insertion of the uterine manipulator | Failed to insert the manipulator or done with trauma to tissues |  | Inserted adequately, but with difficulty |  | Performed smoothly and skillfully | /5 |  |
|  |  | 5.4 | Check that uterine manipulator allows appropriate exposure (i.e.: it has its 6 degrees of freedom) | Not performed |  | Not all directions checked |  | Performed adequately | /5 |  |
|  |  | 5.5 | Check access to pouch of Douglas and sub-ovarian fossas | Not performed |  | Performed after delay without mobilizing manipulator |  | Performed adequately | /5 |  |
|  |  | 5.6 | Check ureter's path in the pelvis | Not performed |  | Position not clearly established, only one ureter identified |  | Identified clearly and skillfully | /5 |  |
| **6** | **Division of the round ligaments (left & right)** | 6.1 | Manipulator: push uterus cranially and laterally towards the opposite side | Not performed |  | Uterus mobilized, but suboptimal exposure |  | Performed adequately, allowing optimal exposure | /10 | /30 |
|  |  | 6.2 | Coagulation and transection of the round ligament | Performed to close to uterine horn; caused significant bleeding or tissue trauma |  | Performed adequately, with minor bleeding |  | Performed skillfully and efficiently | /10 |  |
|  |  |  |  |  |  |  |  |  |  |  |
|  |  | 6.3 | Individualize the front and back fold of the anterior leaf of the broad ligament | Not performed |  | Performed adequately, but the plan was not easily found |  | Plan developed skilfully with minimal bleeding and tissue trauma | /10 |  |
|  |  |  |  |  |  |  |  |  |  |  |
| **7** | **Division of IP ligament or utero-ovarian ligament (left & right)** | 7.1 | Manipulator: push uterus cranially and laterally towards the opposite side | Not performed |  | Uterus mobilized, but suboptimal exposure |  | Performed adequately allowing optimal exposure | /10 | /50 |
|  |  | 7.2 | Expose IP ligament or utero-ovarian ligament | Inadequate exposure putting surrounding structures at risk |  | Performed adequately |  | Performed adequately, allowing optimal exposure | /10 |  |
|  |  | If fenestration is performed | 7.3 Expose the posterior leaf of the broad ligament in its grey area | Unable to expose the posterior leaf |  | Performed adequately, with minor bleeding |  | Performed skillfully and efficiently | /10 |  |
|  |  |  | 7.4 Open a peritoneal window in the broad ligament and enlarge | Performed inadequately: did not check underlying structures or caused damage |  | Performed adequately, in the right direction, with minor bleeding |  | Performed skillfully and efficiently, peritoneal window enlarged by divergent traction |  |  |
|  |  |  | 7.5 Check the ureter has been put at a distance | Not performed |  | Position not clearly established |  | Clearly identified |  |  |
|  |  | If fenestration is not performed: | 7.6 Identify the ureter by transperitoneal visualization | Not performed |  | Position not clearly established |  | Clearly identified | /10 |  |
|  |  |  |  |  |  |  |  |  |  |  |
|  |  | 7.7 | Coagulate using an appropriate energy source or suture | Coagulated too close to surrounding structures or insufficiently |  | Coagulated at the appropriate level, with some hesitation |  | Coagulated safely and efficiently at a 90° angle | /10 |  |
|  |  | 7.8 | Section the IP ligament or the utero-ovarian ligament | Section causing significant bleeding |  | Section causing minor bleeding rapidly controlled |  | Performed skillfully at a 90° angle, causing no bleeding | /10 |  |
| **8** | **Creation of the bladder flap** | 8.1 | Manipulator: push uterus cranially and laterally to the opposite side (left and right) | Not performed |  | Uterus mobilized but required repositioning, suboptimal exposure |  | Performed adequately allowing optimal exposure | /10 | /40 |
|  |  | 8.2 | Open the anterior fold of the broad ligament on both sides down to the level of the vesico-uterine reflection | Performed inadequately: in the wrong plan, causing excessive bleeding, in the wrong direction |  | Performed clumsily but in the right plan. Slow to control bleeding |  | Performed smoothly in the right plan | /10 |  |
|  |  | 8.3 | Manipulator: push uterus cranially and posteriorly | Not performed |  | Uterus mobilized but required repositioning, suboptimal exposure |  | Performed adequately allowing optimal exposure | /5 |  |
|  |  | 8.4 | Section the peritoneum down to the lower uterine segment | Not performed, or caused significant bleeding or tissue trauma |  | Performed adequately, with hesitation |  | Performed adequately in the right plan, with no bleeding | /5 |  |
|  |  | 8.5 | The bladder is grasped at the midline, applying an anterior-superior traction | Not performed |  | Performed with insufficient traction or bladder not properly grasped |  | Grasped with an atraumatic forceps applying the right amount of traction | /5 |  |
|  |  |  |  |  |  |  |  |  |  |  |
|  |  | 8.6 | Opening of the vesico-uterine space at the midline until the cervico-vaginal margin is exposed | Opening in the wrong plan, caused significant bleeding or tissue trauma |  | Moderate difficulty to identify correct plan, occasional bleeding |  | Opening in the correct plan down to the cervico-vaginal margin; no bleeding | /5 |  |
| **9** | **Opening of the posterior peritoneum (left & right)** | 9.1 | Manipulator: push uterus anteriorly and cranially towards the opposite side | Not performed |  | Uterus mobilized but required repositioning, suboptimal exposure |  | Performed adequately allowing optimal exposure | /10 | /20 |
|  |  | 9.2 | Dissection and section of the posterior leaf of the broad ligament towards the insertion of the utero-sacral ligaments on each side | Performed in the wrong direction or in the wrong plan. caused significant bleeding |  | Performed adequately (Moderate difficulty to identify correct plan, occasional bleeding) |  | Performed smoothly and skillfully | /10 |  |
| **10** | **Division of the uterine vessels (left & right)** | 10.1 | Manipulator: push uterus cranially and to the opposite side | Not performed |  | Uterus mobilized but required repositioning, suboptimal exposure |  | Performed adequately allowing optimal exposure | /10 | /60 |
|  |  | 10.2 | Optimize exposure of the uterine vessels | Not performed |  | Insufficient exposure |  | Excellent exposure (retracting fundus in the opposite direction) | /10 |  |
|  |  | 10.3 | Skeletonize uterine vessels at the ascending portion of the uterine artery | Insufficient dissection of uterine artery; caused bleeding or tissue damage |  | Sufficient exposure of uterine artery but done with difficulty or causing moderate bleeding |  | Uterine vessels perfectly exposed, no bleeding | /10 |  |
|  |  | 10.4 | If anatomy is distorted: Identify the ureter prior to division of the uterine vessels | Not performed |  | Position not clearly identified |  | Position clearly identified | /- 10  or  NA |  |
|  |  | 10.5 | Coagulate the uterine vessels using an appropriate energy source or suture | Performed inadequately: at the wrong level, incorrect angle or inappropriate energy source. |  | Performed adequately but with hesitation |  | Performed skillfully and efficiently | /10 |  |
|  |  |  |  |  |  |  |  |  |  |  |
|  |  | 10.6 | Section uterine vessels in the ascending portion, at the level of the colpotomizer | Section at the wrong level occasioning important bleeding or tissue trauma |  | Performed adequately but with hesitation; occasioned bleeding but was able to control |  | Performed efficiently and skillfully, at a 90° angle | /10 |  |
|  |  | 10.7 | Divide cervical attachments of the cardinal ligaments | Not performed, or peformed with an incorrect angle, or causing significant bleeding or tissue trauma |  | Performed efficiently but required some time |  | Performed skillfully and efficiently | /10 |  |
|  |  |  |  |  |  |  |  |  |  |  |
|  |  |  |  |  |  |  |  |  |  |  |
| **11** | **Colpotomy** | 11.1 | Manipulator: push uterus cranially | Not performed |  | Uterus mobilized but required repositioning, suboptimal exposure |  | Performed adequately allowing optimal exposure | /5 | /20 |
|  |  | 11.2 | Identify the cervico-vaginal delineation from the colpotomizer | Cervico-vaginal delineation poorly identified |  | Identified with some difficulty or incompletely |  | Cervico-vaginal delineation clearly identified | /5 |  |
|  |  |  |  |  |  |  |  |  |  |  |
|  |  | 11.3 | Check that there are no interposed elements around the vaginal fornices and complete dissection if necessary | Not performed |  | Performed partially and hastily |  | Careful inspection, clear visualization of the distance from bladder, rectum and ureters | /5 |  |
|  |  | 11.4 | Proceed to circumferential colpotomy using an appropriate energy source | Performed in wrong location, not over the colpotomizer; caused excessive bleeding or tissue trauma; poor exposure |  | Performed adequately but with difficulty, causing moderate bleeding |  | Performed skillfully over the colpotomizer on the full circumference of the vaginal fornices | /5 |  |
| **12** | **Uterus retrieval and vault closure** | 12.1 | Specimen retrieval vaginally | Failed to perform or caused tissue damage |  | Performed adequately but with difficulty |  | Performed efficiently and safely (checking integrity of surrounding structures) | /5 | /20 |
|  |  | **If the specimen is not retrieved in once piece through vaginal route, this step will not be assessed** | | | | | | | |  |
|  |  | 12.2 | Occlude vagina to restore pneumoperitoneum | Looses pneumoperitoneum entirely |  | Performed after delay or partial leak of pneumoperitoneum which is not corrected |  | Pneumoperitoneum fully restored, performed rapidly allowing good exposure | /5 |  |
|  |  | 12.3 | Suture the vaginal vault with interrupted or continuous sutures | Inadequate knot tying technique, damage to surrounding structures or poor quality of suture |  | Performed adequately, tissues generally well approximated |  | Performed skillfully and smoothly; Tissues very well approximated | /5 |  |
|  |  | 12.4 | Vaginal suture including sufficient width of vaginal mucosa and fascia | Suture not including both layers; Insufficient bites |  | Performed adequately, but uneven width of vagina |  | Performed efficiently with regular good bites | /5 |  |
|  |  | 12.5 | Suture includes US ligaments for pelvic support ( if US insertion divided from the vagina during colpotomy ) | Not performed, or caused damage to surrounding structures |  | Performed adequately and clumsily |  | Performed skillfully and efficiently | /- 5 or NA |  |
| **13** | **Hemostasis and inspection** | 13.1 | Irrigation and aspiration of the pelvis | Not performed |  | Performed incompletely or required some time to perform |  | Performed skillfully and efficiently | /5 | /15 |
|  |  | 13.2 | Check vascular pedicles, bladder reflection and vaginal cuff under low abdominal pressure: secure hemostasis if needed | Not performed |  | Performed adequately but not all areas inspected |  | Careful inspection of all areas and meticulous hemostasis if needed | /5 |  |
|  |  |  |  |  |  |  |  |  |  |  |
|  |  | 13.3 | Check there is no damage to surrounding structures | Not performed |  | Moderately detailed inspection |  | Careful and thorough inspection | /5 |  |
|  |  | 13.4 | Perform cystoscopy or Indigo Carmin test if ureteral integrity is of concern. | - 5 if not performed | | | | | NA  or  -5 |  |
|  |  | 13.5 | Perform cystoscopy or bleu test if bladder integrity is of concern. | - 5 if not performed | | | | | NA  or  -5 |  |
| **14** | **Port removal and skin closure** | 14.1 | Remove trocars under direct visualization and inspect port sites for hemostasis | Not performed |  | Performed incompletely |  | Performed smoothly and skillfully | /5 | /20 |
|  |  | 14.2 | Evacuate pneumoperitoneum | Not performed |  | Incompletely evacuated |  | Completely evacuated | /5 |  |
|  |  | 14.3 | Suture fascia for trocars ≥ 10 mm | Not performed or inadequately |  | Required some time to perform but performed correctly |  | Performed efficiently and skillfully | /5 |  |
|  |  | 14.4 | Close skin incisions with any acceptable method | Performed inadequately |  | Required some time to perform but performed correctly |  | Performed efficiently and skillfully | /5 |  |
| **15** | **Order** | 15.1 | Perform tasks 1 (patient positioning) to 5 (inspection of the pelvis) in a chronological order |  | | | | | /5 | /10 |
|  |  | 15.2 | Perform tasks 10 (division of the uterine vessels) to 14 (port removal) in a chronological order |  | | | | | /5 |  |
|  |  | 15.3 | Task 11 (colpotomy) started before task 10 (division of the uterine vessels) | - 5 if colpotomy started before division of the uterine vessels completed | | | | | NA  or  -5 |  |
